# Water deficit affects inter‐kingdom microbial connections in plant rhizosphere

**DOI:** 10.1111/1462-2920.16031

**Published:** 2022-05-17

**Authors:** Kathryn E. Bazany, Jun‐Tao Wang, Manuel Delgado‐Baquerizo, Brajesh K. Singh, Pankaj Trivedi

**Affiliations:** ^1^ Microbiome Network and Department of Agricultural Biology Colorado State University Fort Collins CO; ^2^ Hawkesbury Institute for the Environment Western Sydney University Penrith NSW 2751 Australia; ^3^ State Key Laboratory of Urban and Regional Ecology, Research Center for Eco‐Environmental Sciences Chinese Academy of Sciences Beijing 100085 China; ^4^ Laboratorio de Biodiversidad y Funcionamiento Ecosistémico Instituto de Recursos Naturales y Agrobiología de Sevilla (IRNAS), CSIC Av. Reina Mercedes 10 E‐41012 Sevilla Spain; ^5^ Unidad Asociada CSIC‐UPO (BioFun) Universidad Pablo de Olavide 41013 Sevilla Spain; ^6^ Global Centre for Land‐Based Innovation Western Sydney University Penrith NSW 2751 Australia

## Abstract

The frequency and severity of drought are increasing due to anthropogenic climate change and are already limiting cropping system productivity in many regions around the world. Few microbial groups within plant microbiomes can potentially contribute towards the fitness and productivity of their hosts under abiotic stress events including water deficits. However, microbial communities are complex and integrative work considering the multiple co‐existing groups of organisms is needed to better understand how the entire microbiome responds to environmental stresses. We hypothesize that water deficit stress will differentially shape bacterial, fungal, and protistan microbiome composition and influence inter‐kingdom microbial interactions in the rhizospheres of corn and sugar beet. We used amplicon sequencing to profile bacterial, fungal, and protistan communities in corn and sugar beet rhizospheres grown under irrigated and water deficit conditions. The water deficit treatment had a stronger influence than host species on bacterial composition, whereas the opposite was true for protists. These results indicate that different microbial kingdoms have variable responses to environmental stress and host factors. Water deficit also influenced intra‐ and inter‐kingdom microbial associations, wherein the protist taxa formed a separate cluster under water deficit conditions. Our findings help elucidate the influence of environmental and host drivers of bacterial, fungal, and protistan community assembly and co‐occurrence in agricultural rhizosphere environments.

## Introduction

Climate change will have a substantial influence on agriculture as it will increase water demand, limit agricultural production, and exacerbate water scarcity. To be economically and agronomically viable, crop plants need to remain productive in water deficit (WD) environments, which are likely to become more frequent and intense in the future due to climate change (Cotter and Reyes, 2008). The ability of crops to face environmental stresses such as water scarcity can be partly mitigated by the microbiome inhabiting the soil, rhizosphere, roots, and other plants compartments (Naylor and Coleman‐Derr, 2018; de Vries *et al*., 2020; Trivedi *et al*., 2020; Trivedi *et al*., 2022). Elucidating the dynamic relationships between soil microbes and plants during stress is essential for predicting and potentially managing plant–microbiome interactions to increase the resilience of crop production to abiotic stresses (Naylor and Coleman‐Derr, 2018; de Vries *et al*., 2020; Trivedi *et al*., 2021; Trivedi *et al*., 2022). However, there is little information on how WD influences the complex microbial interactions supported by plant environments, and the degree to which such changes are conserved across different plant hosts.

WD‐mediated changes in plant physiology and metabolism are reported to influence the structure and function of the plant microbiome with consequences on plant performance and health (Naylor and Coleman‐Derr, 2018; Xu and Coleman‐Derr, 2019; de Vries *et al*., 2020; Santos‐Medellín *et al*., 2021; Trivedi *et al*., 2022). This is likely caused by a combination of top‐down processes in the form of control exerted by the plant on its microbiome and bottom‐up processes, namely the responses of the microbial community to the drought itself (Trivedi *et al*., 2022). The influence of the plant can be explained by the emerging ‘cry for help’ hypothesis that posits that plants intentionally recruit specific microbes that can alleviate plant stress (Rolfe *et al*., 2019; Rodriguez and Durán, 2020; Rizaludin *et al*., 2021). According to this hypothesis, upon perception of water stress, plants adjust their root exudation profiles, releasing exudates that can then serve as selective signals, chemo‐attractants and/or nutritional sources to stimulate beneficial microbial communities to colonize and provide relief (de Vries *et al*., 2020). On the other hand, the microbial recruitment could be a general by‐product mediated by the direct impact of WD on the microbial seed bank or the indirect impact of changes in the plant physiology (Trivedi *et al*., 2022). The complex ways these processes interact to shape the plant microbiome are highly circumstantial and currently not well understood.

Plant‐associated microbial communities form highly complex ecological networks that include multiple associations between co‐existing taxa. Climate extremes can reorganize networks of associations between co‐existing soil microbial taxa (Zhou *et al*., 2011; de Vries *et al*., 2018; Bardgett and Caruso, 2020; Yuan *et al*., 2021) with essential feedback on plant resilience and performance in stress environments. For example, recent studies have shown that WD significantly influences microbial co‐existence networks (de Vries *et al*., 2018; Zhang *et al*. 2021; Xie *et al*., 2021). These changes in the microbial co‐existence network potentially impact the recovery of microbial communities and alter plant–microbe interactions under disturbances. In microbial co‐existence networks, positive and negative associations represent aggregation and exclusion, respectively. Research on microbial co‐existence networks and their topologies suggest that negative association patterns maximize robustness and stability under disturbances (Coyte *et al*., 2015; de Vries *et al*., 2018). While the impact of climate change stressors on the individual microbial networks for certain microbial groups have been studied (de Vries *et al*., 2018), there is limited information on how WD impacts the direction and strength of intra‐kingdom associations across contrasting crops.

In plant environments, trophic interactions are governed by protists that act as top‐down controllers of microbial communities and influence food webs by preying on a wide range of bacteria, fungi, and other eukaryotes (Geisen *et al*., 2018; Gao *et al*., 2019; Sun *et al*., 2021). Protists and their interactions with other microorganisms are also subject to change during environmental stress events (Geisen *et al*., 2018; Gao *et al*., 2019). Studies have shown that trophic cascades can be destabilized by strong changes to a few important interactions in a food web (de Vries *et al*., 2018; Bardgett and Caruso, 2020). Furthermore, few studies have shown that protists form central hubs in microbial co‐existence networks, linking diverse bacterial and fungal groups (Xiong *et al*., 2018; Sun *et al*., 2021). Given their key position, protists can amplify or dampen the impact of environmental perturbations on the microbial co‐existence networks in plant‐associated environments. However, compared to bacteria and fungi, the impact of WD on rhizosphere protists has seldom been investigated.

Here, we explored the impact of WD on the diversity, community composition, and associations between multi‐kingdom rhizosphere microbial groups (bacteria, fungi, and protists) of corn and sugar beet. We chose corn and sugar beet for their commercial relevance, ease of sampling, and physiological differences, as corn is a C4 monocot and sugar beet is a C3 dicot. We tested the following hypotheses. First, we hypothesized that WD will have variable influence on the rhizosphere microbial community composition of different microbial groups (bacteria, fungi, and protists). This hypothesis was based on past work showing that the response of microbes to WD can vary based on differences in molecular, cellular, physiological, and morphological traits (Naylor and Coleman‐Derr, 2018; Xu and Coleman‐Derr, 2019; de Vries *et al*., 2020; Trivedi *et al*., 2022). Second, we hypothesized that WD will decrease the connectedness of microbial co‐existence networks by influencing inter‐kingdom associations. This hypothesis was based on past work showing that the changes in the species interactions mediated by environmental disturbances promote destabilizing properties in microbial co‐existence networks (de Vries *et al*., 2018; Bardgett and Caruso, 2020; Hernandez *et al*., 2021).

To test our hypothesis, we collected rhizosphere soil samples from eight sites across four mid‐western states in the United States (Supplementary Table S1). We specifically selected for sites where corn and sugar beet were growing in adjacent fields, and the irrigation was maintained through centre‐pivot (also known as waterwheel and circle irrigation). Under these conditions a natural moisture gradient can be observed between the inner (>20 rows) and the outer (<15 rows) of planted crops. Thus, here, WD is established by a prolonged reduction in water availability compared with fully irrigated (IR) plants closer to the water source. We aimed to explore the taxonomic differences between the rhizosphere microbiomes of WD and IR plants using amplicon (for bacteria, fungi, and protists) sequencing. We also compared the inter‐kingdom microbial networks of WD and irrigated plant microbiomes to offer insights into the connectedness and proportion of positive/negative interactions, which serves as a measure of network stability; the hubs within the network, which indicates prominent microbial groups; as well as highly correlated taxonomic groups, indicating microbes that tend to co‐exist with one another.

Our results show that microbial co‐existence networks differ significantly in key properties such as network connectivity and inter‐ and intra‐kingdom interactions that might inform on their stability under water stress. We also show that a WD‐induced shift in the microbial community composition varies with different microbial groups with bacteria and protists being more sensitive to water limitation compared to fungi. Disentangling the role of crucial microbial taxa in microbiome communities in WD conditions might provide suitable approaches to harness plant–microbiome interactions to alleviate water stress. Altogether, we provide novel evidence that WD significantly affects microbial community taxonomic composition and co‐occurrence network structures in the rhizosphere, which have implications for the potential changes in their ecological functions under climate change.

## Experimental procedures

### Site selection and sample collection

Eight sugar beet fields with directly adjacent corn fields were identified with help from the Western Sugar Cooperative and selected as sites (Supplementary Table S1). Each site contained two crop fields, one sugar beet and one corn. Each crop field contained two irrigation treatments, irrigated (IR), defined as within the reach of the irrigation machinery of the site, and non‐irrigated or water deficit (WD) stress, defined as crops beyond the reach of the field's irrigation machinery. The yearly average rainfall from the sampling regions ranges from 431 to 533 mm. Under these conditions, farming for corn and sugar beet is not possible without supplemental irrigation. A lack of irrigation exerts a strong abiotic stress on plants in this region, which has a notable impact on plant and productivity that is equivalent to drought in other regions. Three plants from each crop type were collected from each treatment, totalling 12 plants per site. We collected plant samples from 30 to 40 and 8 to 12 rows inside the field representing IR and WD treatments, respectively. Sample collection was conducted in the summer of 2020 at the flowering time for both the plant species. Samples were shipped to the laboratory at Fort Collins on ice.

### Sampling processing and DNA extraction

We used a detailed protocol from Simmons *et al*. (2018) to separate rhizosphere soil samples. Rhizosphere soils were defined as soil clinging tightly to the plant's roots. DNA was extracted from soils using the DNeasy Powersoil Kit (MO BIO Laboratories, Carlsbad, CA, USA) as per manufacturer's instruction. Extracted DNA was quality checked by NanoDrop 2000 (Thermo Fisher Scientific, Waltham, Massachusetts, USA), quantity checked by Qubit Fluorometer (Thermo Fisher Scientific), and stored at −80 °C.

### Measurement of soil physicochemical properties

Soil properties were determined following conventional methods. Soil gravimetric water content was determined by weighing 5 g of fresh, sieved composite sample, oven drying, and reweighing after no further mass loss. Soil organic carbon (OC) was determined by the combustion method on an element analyser using air‐dried soils (Vario MAX C/N, Germany) pH was measured using a pH metre, in a 1:2.5 mass:volume suspension of soil and water.

### Amplicon sequencing and bioinformatic analyses

The diversity and community structure of soil bacteria, fungi, protists, and invertebrates was determined by amplicon sequencing using an Illumina MiSeq platform. We used the primer sets 515F/806R (Caporaso *et al*., 2012), ITS1F/ITS2R (Caporaso *et al*., 2012) and Euk1391f/EukBr (Amaral‐Zettler *et al*., 2009; Stoeck *et al*., 2010) to amplify a portion of the bacterial 16S rRNA gene, fungal ITS1 region, and the eukaryotic 18S rRNA gene, respectively. Bioinformatics processing was performed using a combination of USEARCH (Edgar, 2010) and UNOISE3 (Edgar, 2016). Amplicon sequence variant (ASV) tables based on 97% sequence similarity were generated using the USEARCH pipeline. Sequencing run quality was assessed using fastQC (Andrews, 2010). The raw sequences were discarded if they contained ambiguous nucleotides, had a low (*Q* < 20) quality score, or were short in length (<100 bp). Adapters and primers were removed using cutadapt (Martin, 2011). Then samples were demultiplexed. Paired‐end reads were merged, and quality was assessed with an initial quality check test. The representative set database was created using the UCLUST and UPARSE algorithm (Edgar, 2013). Unique sequences were located and sorted into unique ASVs. ASVs were clustered using DADA2 and DeNoised using uNoise3 (Xiong *et al*., 2021a) as described (Xiong *et al*., 2021b). ASV tables were generated by mapping reads to the representative set database. ASVs were counted at the sample level. Protistan sequences based on the eukaryotic 18S rRNA gene data were taxonomically assigned against the Protist Ribosomal Reference (PR2) database (Guillou *et al*., 2012). Protists were defined as all eukaryotic taxa, except fungi, invertebrates (Metazoa) and vascular plants (Streptophyta) (Delgado‐Baquerizo *et al*., 2020). Taxonomic identification of bacteria and fungi was obtained against the Silva (Pruesse *et al*., 2007) and UNITE database (Nilsson *et al*., 2019), respectively. Bacterial sequences that match host mitochondria and chloroplast were removed.

### Statistical analysis

Samples were evaluated separately for bacterial (16S), fungal (ITS), and protistan (18S) communities. Samples were rarified to the lowest occupancy of 8000, 5000, and 3300 reads for 16S rRNA, ITS, and 18S rRNA, respectively. We used the R package ‘mctools’ to analyse microbial community structure (Leff, 2017). To examine beta diversity, Bray–Curtis dissimilarity distances were calculated then ordinated in multidimensional scaling using a constrained analysis of principal coordinates (CAPs) analysis to irrigation treatments. Permutational multivariate analysis of variance (PERMANOVA) models were generated to determine significant beta‐diversity differences correlating with niche compartment, species, site, and irrigation treatment. To examine alpha diversity, Shannon diversity indexes were calculated and evaluated through general linear models (GLMs). Tukey HSD tests were used to determine influence of the above variables on alpha‐diversity. To investigate the indicator taxa involved in the differences between IR and WD community, a linear discriminate analysis (LDA) effect size (LEfSe) was conducted to explore the differential microbial populations at the phylum level for bacteria and family level for fungi and protists (Segata *et al*., 2011). A significance level of α ≤ 0.05 was used for all biomarkers evaluated in this study. All statistical analyses were completed using R v 4.0.5 (R Core Team, 2020).

Structure equation model (SEM) was used to analyse the relationships among soil water content, soil properties (pH, OC, moisture), microbial alpha and beta‐diversity for both corn and sugar beet (Trivedi *et al*., 2016; Trivedi *et al*., 2017; Ochoa‐Hueso *et al*., 2018). CAP1 and CAP2 were used to proxy the variance of microbial community composition. Shannon diversity indexes were used as a proxy for alpha‐diversity. The *a priori* models included all possible pathways among these factors. The significance of each path‐coefficient was analysed by calculating its critical ratio (*P* < 0.05). The overall model fit was evaluated with the Bentler comparative fit index, goodness‐of‐fit index and chi‐square test (Trivedi *et al*., 2016; Trivedi *et al*., 2017; Ochoa‐Hueso *et al*., 2018). The SEM was performed using Amos Graphics v22 (IBM Corp., Armonk, NY, USA).

### Microbial correlation networks

Correlations among bacterial, fungal and protists ASVs were calculated to generate a co‐existing network of the three groups. To minimize the influence of rare taxa, only ASVs with more than five reads and three observations were kept in the calculation. We controlled the false discovery rate by performing 1000 bootstraps on each correlation. We kept only the strong (*r* > 0.60) and robust (*P* < 0.01) correlations. The network calculation was performed using the SparCC‐based (Friedman and Alm, 2012) algorithm Fastspar (Watts *et al*., 2019). The networks were displayed in the software Gephi (Bastian *et al*., 2009). Topological properties including nodes, edges numbers, degree, and Closeness centrality and between centrality were also calculated in Gephi. Scatter plots were generated using the ggplot2 package in R platform.

## Results and discussion

### Water deficit impacts the structure of rhizosphere microbiota

Our study shows that inter‐kingdom microbial connections in plant rhizospheres are highly sensitive to WD stress in two contrasting crops; however, we also found that such impacts are soil taxa and site dependent. Our soil dataset showed significant (30%–63%) reductions in the moisture content in the WD compared to the irrigated (IR) treatments for both corn and sugar beet within each site (Supplementary Fig. S1). The reduction in soil moisture in our study is similar to that in controlled greenhouse experiments designed to compare the impact of WD vs well‐watered conditions on plant performance (Puértolas *et al*., 2017; Singh *et al*., 2021). Our analyses also showed mostly non‐significant difference in pH and organic carbon (OC) in WD and IR treatments for corn and sugar beet within each site (Supplementary Figs. S2 and S3).

**Fig. 1:**
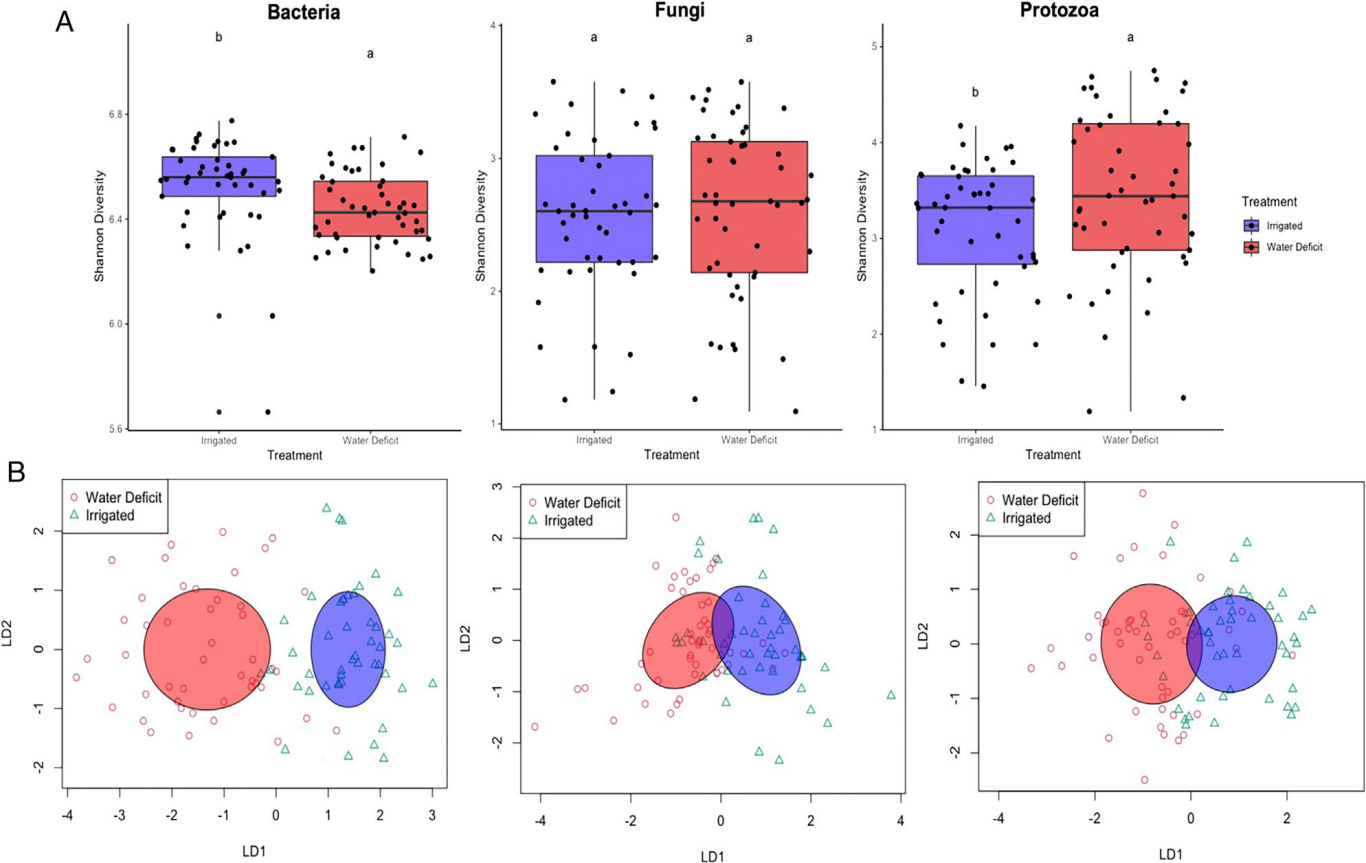
Impact of water deficit on the alpha (A) and beta (B) diversity of bacteria, fungi, and protists. A. Differences between Shannon diversity of bacteria, fungi, and protists in treatments. The boxes show the average Shannon diversity of corn and sugar beet rhizosphere under irrigated and water deficit treatment for bacteria, fungi, and protists. Different letters indicate statistically significant differences (*P* < 0.05). B. Ordination biplots for canonical analysis of principal coordinates (CAP) illustrating the impact of water deficit (red colour circles) and irrigation (green coloured triangles) on the combined rhizosphere bacteria, fungi, and protists community of corn and sugar beet. CAP analysis is based on the Bray–Curtis dissimilarity.

Using general linear models (GLMs), we found that the impact of WD on alpha‐diversity was significant for bacteria (*P* < 0.005) and protists (*P* < 0.05) but not for fungi (Fig. 1A). The alpha diversity of bacteria decreased in WD as compared to IR treatments while the opposite trend was observed for protists (Fig. 1A). Moreover, crop species were the most important driver of alpha diversity for fungi and protists (*P* < 0.005) but for bacteria, the influence of crop species was non‐significant. The interaction between species:site:treatment was significant for bacteria (*P* < 0.005) and protists (*P* < 0.05) but non‐significant for fungi (Table 1). Our results are in lines with those in de Vries *et al*. (2018) suggesting that fungal communities are usually resistant to water stress.

**Table 1:** The effects of plant species, site, treatment, and their interactions on the changes of alpha diversity of bacterial, fungal, and protistan communities based on linear mixed model (LMM).

	Bacteria		Fungi		Protist	
Drivers	*F*‐value	*P* value	*F*‐value	*P* value	*F*‐value	*P* value
Site	5.187	0.0001165***	17.9972	0.001*, **	2.55	0.022*
Treatment	9.645	0.0028979**	0.2203	0.64	3.894	0.053.
Species	0.3181	0.5748754	17.3878	0.001***	14.838	0.001***
Site:Treatment	0.729	0.6480487	1.0568	0.402	1.25	0.289
Site:Species	2.273	0.0402901*	4.9451	0.0001***	1.075	0.39
Treatment:Species	3.7906	0.0562256	0.1262	0.724	10.124	0.002**
Site:Treatment:Species	4.1175	0.0009398***	1.1797	0.327	2.164	0.049*

* *P* < 0.05.

** *P* < 0.005.

*** *P* < 0.001.

Our results align with the findings of Schmitt and Glaser (2011) who reported that water limitation increased protistan diversity. Protists are primarily aquatic and therefore it is presumed that they will be sensitive to WD (Harder *et al*., 2016). However, protistan taxa have a variety of lifestyles and body sizes, exhibiting a range of tolerance to soil moisture conditions (Stefan *et al*., 2014; Fierer, 2017; Geisen *et al*., 2018). Our results also are in accordance with an earlier study that reported that protist community composition and dynamics are filtered by the influence of plants on their rhizosphere biological and physicochemical environment, resulting in similar patterns observed for rhizosphere bacterial communities (Ceja‐Navarro *et al*., 2021).

We further assessed the relative contribution of multiple factors in terms of sampling sites, plant species, and WD in shaping the rhizosphere microbial communities. PERMANOVA analysis revealed that all examined drivers and their interactions have a significant impact (*P* < 0.005) on the structure of bacterial, fungal, and protistan communities (Table 2). As a result of differing environmental conditions (e.g. sites, soil moisture content), shifts in microbial community composition are driven by changes in relative abundance of microbial species, rather than complete disappearance, which explains the importance of all the studied factors in driving beta but not alpha‐diversity (Naylor and Coleman‐Derr, 2018). The greatest effect on the total microbiome was exerted by the sampling site (*R* ^2^ = 0.47 for bacteria; *R* ^2^ = 0.53 for fungi, and *R* ^2^ = 0.39 for protist; *P* < 0.001 for all three). Our sampling sites varied in soil properties including pH and organic C, both of which are reported to be the major drivers for microbial community composition (Trivedi *et al*., 2016; Fierer, 2017; Ochoa‐Hueso *et al*., 2018). The sampling site effect represented the interaction effect of site‐dependent environmental characteristics (e.g. climate and soil type) and has been shown as the major driver co‐influencing the microbiome composition (Coleman‐Derr *et al*., 2016; Santos‐Medellín *et al*., 2017; Hamonts *et al*., 2018; Guo *et al*., 2021).

**Table 2:** PERMANOVA output showing importance of plant species, site, treatment, and their interactions as factors shaping the microbial community of bacteria, fungi, and protists.

	Bacteria			Fungi			Protist		
	*F*‐value	*R* ^2^	*P* value	*F*‐value	*R* ^2^	*P* value	*F*‐value	*R* ^2^	*P* value
Species	4.394	0.0185	***	9.65	0.0281	***	22.822	0.07965	***
Site	15.805	0.46576	***	25.135	0.51244	***	16.133	0.39416	***
Treatment	9.856	0.04149	***	8.172	0.0238	***	9.769	0.0341	***
Species:Site	3.348	0.09867	***	7.545	0.15383	***	5.738	0.14019	***
Species:Treatment	2.111	0.00889	**	3.33	0.0097	**	7.175	0.02504	***
Site:Treatment	2.269	0.06686	***	2.531	0.05159	***	2.329	0.0569	***
Species:Site:Treatment	1.603	0.04725	***	1.96	0.03995	**	1.907	0.04658	***
Residuals		0.2526			0.18058			0.22338	

*** *P* < 0.001.

** *P* < 0.005.

WD was an important driver for all the three groups of microbial communities (*R* ^2^ = 0.041 for bacteria; *R* ^2^ = 0.023 for fungi, and *R* ^2^ = 0.034 for protists; *P* < 0.001 for all three) (Table 1, Fig. 1B). Few studies have reported small or non‐existent impacts of water limitation on soil or root fungal communities (Yuste *et al*., 2011; Bouasria *et al*., 2012; Fuchslueger *et al*., 2016). Our study observed that although the variation explained by WD on bacteria and protists was more significant than on fungi, the impact of WD on fungal communities was still significant. Fungal hyphae networks can allow remote access and redistribution of water that can improve host tolerance towards WD. It is reported that plant presence can modify the impact of water limitation on fungal communities, wherein significant variation was observed in rhizosphere and plant compartments but not in bulk soils (Veach *et al*., 2020).

Bacterial communities are reported to be more sensitive to WD than fungal communities (Naylor and Coleman‐Derr 2018; de Vries *et al*., 2018). Bacteria and fungi differ in body size, diversity, metabolic activity, dispersal potential, and in their nature of interaction with host or other microbes, affecting species sorting and the community assembly process under environmental stresses. Interestingly, for bacterial communities, the variation explained by the WD was higher than crop species (*R* ^2^ = 0.018 and 0.041 for species and WD, respectively). Similar results were obtained by Santos‐Medellín *et al*. (2021), wherein they reported that WD explains more bacterial community variation in race than host genotype. Our results thus suggest that WD weakens the correlation between the host phylogeny and bacterial community composition.

**Fig. 2:**
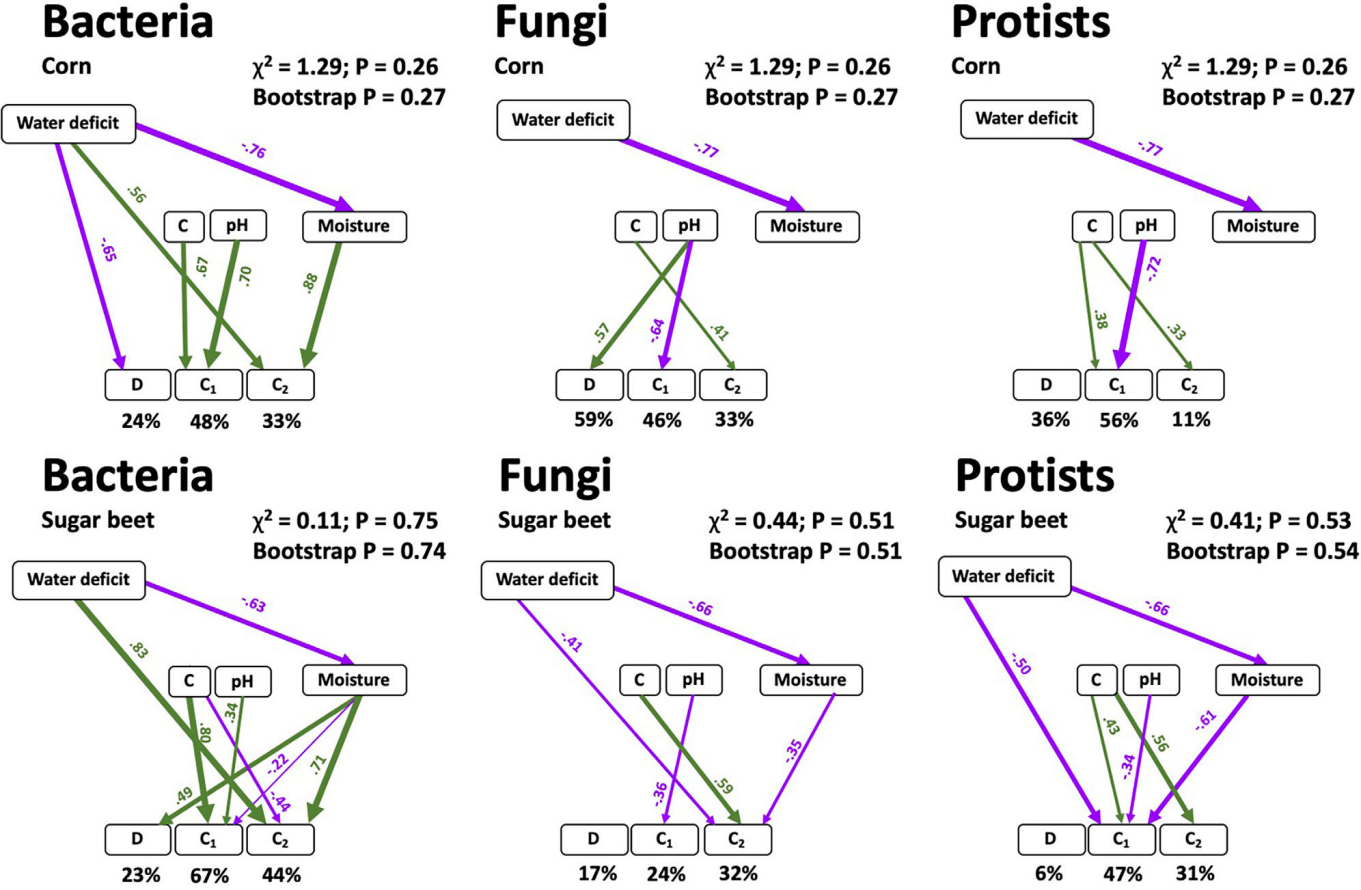
Structural equation models showing the effects of organic carbon (OC), pH, soil moisture and water deficit treatment on the community composition (C1 and C2) and Shannon diversity of bacteria, fungi, and protists in the rhizosphere of corn and sugar beet. Numbers adjacent to arrows are standardized path coefficients, analogous to partial regression weights and indicative of the effect size of the relationship. Arrow width is proportional to the strength of path coefficients. Green and purple arrows represent positive and negative effects, respectively. Model fitness details (*χ* ^2^ and non‐parametric Bootstrap parameters) are close to each figure.

We then used structural equation models (SEMs) to explore the direct and indirect relationships among WD, soil properties – including organic carbon (OC), pH, moisture – and microbial community composition and alpha diversity (Fig. 2). Our SEM showed that WD has a significant negative impact on soil moisture but does not affect OC and pH (Fig. 2; Supplementary Fig. S4). For bacteria, WD had a significant impact on the community composition for both corn and sugar beet. While WD directly impacts bacterial alpha‐diversity in corn rhizospheres, the impact is indirectly mediated though moisture in sugar beet. For corn, we did not observe any impact of WD or moisture on the fungal communities. For sugar beet, our SEM showed a minor impact of both WD and moisture on the second CAP component. These results are in line with other studies suggesting that water stress has a more pronounced impact on bacteria than fungal communities (Naylor and Coleman‐Derr, 2018; de Vries *et al*., 2018). For protists, our SEM analysis did not reveal a significant impact of WD and soil moisture on community composition or diversity in corn. For sugar beet, both WD and soil moisture impact the community composition of protists but not the diversity. Interestingly, while the impact of WD on the bacterial community composition was positive, SEM analysis revealed a significant negative impact of WD on fungal and protistan community.

### Water deficit impacts the enrichment of selected microbial groups in the rhizosphere

**Fig. 3:**
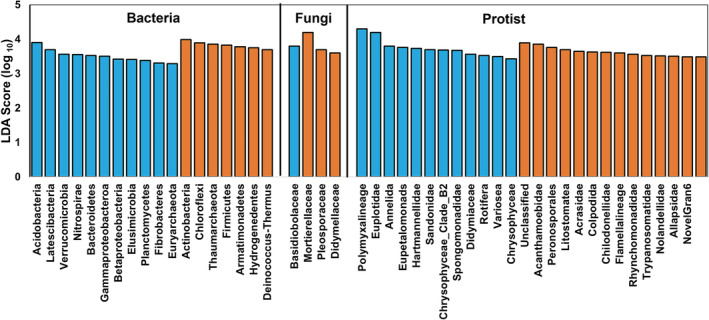
Linear discriminate analysis effect size (LEfSe) analysis of bacterial (phylum), fungal (family), protistan (family) groups that were indicators for irrigated (blue) and water deficit (brown) treatments (LDA score > 3).

We used linear discriminant analysis (LDA) effect size (LEfSe) to compare microbial communities and identify specific phylotypes of corn and sugar beet rhizosphere responding to WD (Fig. 3). We observed that the microbial groups that responded to WD are similar for both the plant species. Generally, bacteria from the phyla Actinobacteria, Firmicutes, Chloroflexi, Deinococcus Thermus, Aramatimonadetes increased in WD while those belonging to the phyla Acidobacteria, Verrucomicrobia, Nitrospirae, Planctomycetes, Euryarchaeota, and class Gammaproteobacteria and Betaproteobacteria were depleted in relative abundance. Our results support the core response to water limitation at phylum level with a universal enrichment of monoderm (Gram‐positive) bacteria and a depletion of most diderm (or Gram‐negative) lineages (Naylor *et al*., 2017; Santos‐Medellín *et al*., 2017, 2021; Fitzpatrick *et al*., 2018; Naylor and Coleman‐Derr, 2018; Xu *et al*., 2018; Xu and Coleman‐Derr, 2019). Resistance against water limitation involves deeply conserved traits such as osmolyte production, cell wall features or spore formation, which are present in similar groups within soil microbial communities, indicating that the context of a particular location does not affect the phylogenetic pattern of response.

Our results provide support for ‘cry for help’ hypothesis. First line of support comes from the selective enrichment of microbial groups particularly monoderm under water deficit conditions. The enrichment of the monoderm is driven in part by the interaction within the plant host and not just on the ability of monoderm to withstand water limitation (Fitzpatrick *et al*., 2018; Naylor and Coleman‐Derr, 2018; Xu *et al*., 2018; Xu and Coleman‐Derr, 2019; Santos‐Medellín *et al*., 2021). For example, under drought stress plants secrete glyceraldehyde‐3‐phosphate, which can be efficiently transported and utilized by Actinobacteria (Xu *et al*., 2018; Xu and Coleman‐Derr, 2019). Furthermore, many monoderm strains are reported to provide drought resistance to several crop plants (Xu *et al*., 2018; Santos‐Medellín *et al*., 2021).

For fungi, we observed only four groups at the family level that were indicators for the IR or WD treatments. This observation again suggests that fungi are more resistant to water limitation than bacteria. Our results showed an enrichment of members of the phyla Mortierellomycotaand depletion of members of Basidiobolomycota in WD compared to IR treatment. Members within both these groups are relatively less dominant and diverse with limited information on the traits that can be related to their drought response.

In comparison to fungi, there were more protist groups that responded to IR or WD. Soil moisture has been reported as the most influential edaphic factor differentially affecting different functional groups within protists (Fiore‐Donno *et al*., 2019). Canarini *et al*. (2021) identified protists as the biomarker for drought along with the Gram‐negative and Gram‐positive bacteria. Members of WD indicator protists families, such as Acanthamoebidae and Flamella lineage, produce cysts that are very efficient in preserving protists for weeks and even years against environmental stresses such as drought (Geisen *et al*., 2018). Members of family Litostomatea, Acrasidae and Allapsidae were identified as WD indicators in our study and are reported to prefer dry environments (Oliverio *et al*., 2020). Overall, we have identified protistan families that are robust bioindicators for WD or IR treatments. Given the variety of functional trophic roles that protists play in shaping microbial dynamics (Bates *et al*., 2013; Gao *et al*., 2019; Sun *et al*., 2021) these bioindicators will be key to understand the trophic complexity in response to water stress.

### Water deficit affects rhizosphere microbiome co‐existence networks

**Fig. 4:**
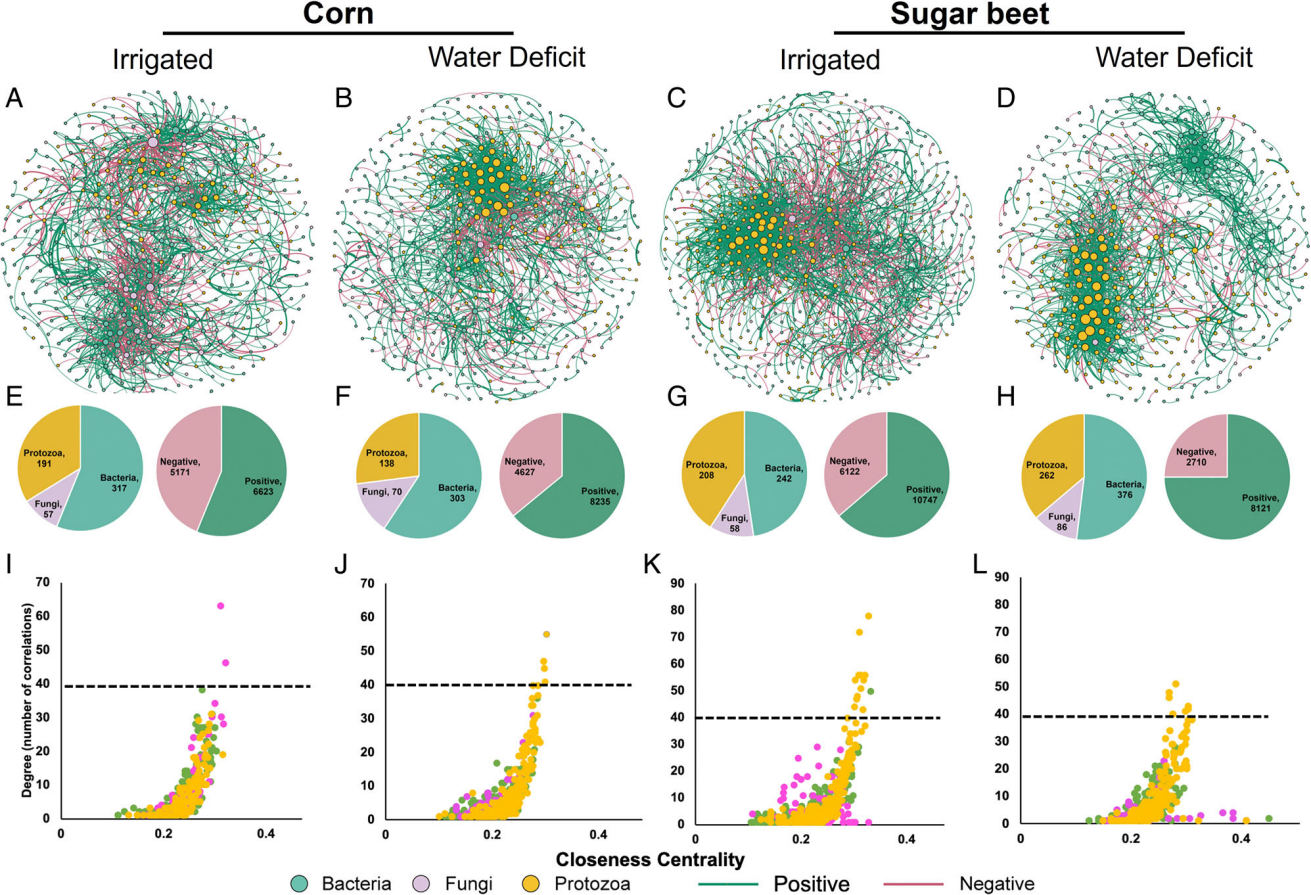
Co‐existing networks of soil bacteria, fungi and protists under different treatments (A – Irrigated Corn; B – Water Deficit Corn; C – Irrigated Sugar Beet; d‐ Water Deficit Sugar Beet). Nodes indicate microbial ASVs (green – bacteria, purple – fungi, yellow – protists) and edges indicate strong (*r* > 0.60) and significant (*P* < 0.01) correlations among ASVs (green edges indicate positive correlation and red edges indicate negative correlation). Under each network, the left pie chart indicates the number of ASVs from each group in individual network, and the right pie chart indicates the number of correlations (positive vs. negative) in each network (E–H). The scatter plots below show the importance of nodes in each network (I–L). Higher betweenness centrality indicates a potential connector while higher closeness centrality indicates a potential module hub.

Our results showed that microbial inter‐kingdom network patterns shifted clearly in response to WD for both corn and sugar beet (Fig. 4). We observed that the protist taxa separated distinctly and formed a separate cluster under WD conditions for both corn and sugar beet (Fig. 4A–D). For corn, the hub microbial taxa in IR conditions were fungi and bacteria, whereas in WD conditions, the hubs were all protists (Fig. 4I and J). For sugar beet, the hub microbial taxa in both IR and WD networks were solely protists (Fig. 4K and L). In the IR networks for both corn and sugar beet, bacterial taxa have lower network connectivity (network degree) (5.36 and 6.01 for corn and sugar beet, respectively; *P* < 0.005) as compared to fungi (9.5 and 7.9 for corn and sugar beet, respectively) and protists (6.8 and 9.8 for corn and sugar beet, respectively) (no significant difference between fungi and protists) (Supplementary Fig. S5). In the WD networks for both the plant species, the average number of degrees for protists (9.2 and 9.3 for corn and sugar beet, respectively) was significantly higher (*P* < 0.005) than bacteria (3.5 and 4.7 for corn and sugar beet, respectively) and fungi (5.7 and 3.7 for corn and sugar beet, respectively) (no significant difference between bacteria and fungi). For both the plant species, we observed a significant decrease (*P* < 0.005) in degree for bacteria (3.5 vs 5.3 and 4.7 vs 6.8 for corn and sugar beet, respectively) and fungi (5.8 vs 9.5 and 3.8 vs 7.8 for corn and sugar beet, respectively) in WD as compared to IR network. Our results thus suggest that in WD networks, protists are more central with a higher number of connections, and the relative centrality of bacteria and fungi is lower than in the IR networks.

In microbiome studies, protists have received little attention despite their key role in controlling bacterial and fungal populations (Geisen *et al*., 2018; Guo *et al*., 2021). Protists are sensitive to environmental disturbances, occupy key position in inter‐kingdom microbial networks, and are postulated to enhance microbial mediated functions (Xiong *et al*., 2018). We illustrate the importance of protists as possible top‐down controllers of microbiome community interactions linked to plant stress response. We therefore propose that a holistic microbiome perspective, including bacteria, fungi, and protists, provides the optimal next step in predicting plant performance under water stress.

The percentage of positive correlations increased from 56% to 65% and 63% to 75% in WD as compared to IR network for corn and sugar beet, respectively (Fig. 4E–H). A large proportion of positive links between the interacting members can cause instability in microbial networks. Conversely, higher positive interactions infer that the members respond similarly to environmental fluctuations resulting in positive feedback and co‐oscillations (Coyte *et al*., 2015; de Vries *et al*., 2018). Our results thus suggest that water limitation will destabilize co‐oscillation in communities and will weaken the stability of networks. Notably, inspection of network architecture indicates that in both the IR and WD networks, there were more positive intra‐kingdom as compared to inter‐kingdom correlations (Supplementary Fig. S6). Interestingly, the percent increase of positive associations in WD was driven by an increase in the intra‐kingdom associations while the inter‐kingdom associations became more negative in WD as compared to IR networks (Supplementary Fig. S6). For example, in sugar beet, the bacteria–bacteria positive associations increased from 63% to 94% in the WD as compared to IR networks. On the other hand, the negative associations between bacteria and protists increased from 59% to 80% in WD as compared to IR networks. The competition between microbial groups for the root exudates is postulated to contribute towards negative correlations between bacteria and eukaryotes (Durán *et al*., 2018). Taken together, these results suggest that the detected microbial inter‐kingdom associations in the rhizosphere become more intense and competitive under WD. Further research involving reconstitution experiments to distangle microbiome interactions will reveal mechanisms that govern microbiome assembly in WD. These insights on the complex plant–microbiome interactions will be crucial in the development of targeted and effective microbial amendments that can improve crop fitness and productivity under WD.

## Conclusions

This study advances the understanding of ecological processes that occur in the rhizosphere of crops under water stress. Additionally, our study highlights the importance to consider protist and their associations with other microbes to evaluate the impact of environmental stresses on crop microbiome. We propose that a deeper understanding on how qualitative and quantitative changes in root exudation affect both competition for resources and cooperative relationships in the rhizosphere will illuminate the specific mechanisms underpinning these interactions, including how changes in microbial community interactions in response to shifting environmental regimes impact plant performance. Understanding the dynamics of inter‐kingdom interactions under stressful conditions will provide a way forward to engineering complex crop microbiomes with predictable behaviour and robust outcomes.

## Authors contribution

P.T. conceived and supervised the study. P.T. and K.B. designed the experiments. K.B. performed the experiments. K.B., J.T., M.D.B., P.T., and B.K.S. analysed the data. P.T. and K.B. wrote the manuscript. All authors read and approved the final manuscript.

## Supporting information


**Appendix S1:** Supporting Information

## Data Availability

The raw sequence data related to this study has been submitted in the NCBI Sequence Read Archive under SRA accessions numbers PRJNA822849 (bacterial 16S rRNA reads), PRJNA822822 (protist 1S rRNA reads) and PRJNA822844 (fungal ITS reads).

## References

[emi16031-bib-0001] Amaral‐Zettler, L.A. , McCliment, E.A. , Ducklow, H.W. , and Huse, S.M. (2009) A method for studying protistan diversity using massively parallel sequencing of V9 hypervariable regions of small‐subunit ribosomal RNA genes. PloS One 4: e6372. 19633714 10.1371/journal.pone.0006372 PMC2711349

[emi16031-bib-0002] Andrews, S. (2010) FastQC: a quality control tool for high throughput sequence data. URL http://www.bioinformatics.babraham.ac.uk/projects/fastqc

[emi16031-bib-0003] Bardgett, R.D. , and Caruso, T. (2020) Soil microbial community responses to climate extremes: resistance, resilience and transitions to alternative states. Philos Trans R Soc Lond B 375: 20190112. 31983338 10.1098/rstb.2019.0112 PMC7017770

[emi16031-bib-0004] Bastian, M. , Heymann, S. , and Jacomy, M. (2009) Gephi: an open source software for exploring and manipulating networks. IWSEM 3: 361‐362.

[emi16031-bib-0005] Bates, S.T. , Clemente, J.C. , Flores, G.E. , Walters, W.A. , Parfrey, L.W. , Knight, R. , and Fierer, N. (2013) Global biogeography of highly diverse protistan communities in soil. ISME J 7: 652–659. 23235291 10.1038/ismej.2012.147 PMC3578557

[emi16031-bib-0006] Bouasria, A. , Mustafa, T. , De Bello, F. , Zinger, L. , Lemperiere, G. , Geremia, R.A. , and Choler, P. (2012) Changes in root‐associated microbial communities are determined by species‐specific plant growth responses to stress and disturbance. Eur J Soil Biol 52: 59–66.

[emi16031-bib-0007] Canarini, A. , Schmidt, H. , Fuchslueger, L. , Martin, V. , Herbold, C.W. , Zezula, D. , *et al*. (2021) Ecological memory of recurrent drought modifies soil processes via changes in soil microbial community. Nat Commun 12: 1–4. 34489463 10.1038/s41467-021-25675-4 PMC8421443

[emi16031-bib-0008] Caporaso, J.G. , Lauber, C.L. , Walters, W.A. , Berg‐Lyons, D. , Huntley, J. , Fierer, N. , *et al*. (2012) Ultra‐high‐throughput microbial community analysis on the Illumina HiSeq and MiSeq platforms. ISME J 6: 1621–1624. 22402401 10.1038/ismej.2012.8 PMC3400413

[emi16031-bib-0009] Ceja‐Navarro, J.A. , Wang, Y. , Ning, D. , Arellano, A. , Ramanculova, L. , Yuan, M.M. , *et al*. (2021) Protist diversity and community complexity in the rhizosphere of switchgrass are dynamic as plants develop. Microbiome 9: 1–8. 33910643 10.1186/s40168-021-01042-9 PMC8082632

[emi16031-bib-0010] Coleman‐Derr, D. , Desgarennes, D. , Fonseca‐Garcia, C. , Gross, S. , Clingenpeel, S. , Woyke, T. , *et al*. (2016) Plant compartment and biogeography affect microbiome composition in cultivated and native agave species. New Phytol 209: 798–811. 26467257 10.1111/nph.13697 PMC5057366

[emi16031-bib-0011] Cotter, J. , and Reyes, T. (2008) Food security and climate change: the answer is biodiversity. In A Review of Scientific Publications on Climate Change Adaptation in Agriculture. Exeter: Greenpeace.

[emi16031-bib-0012] Coyte, K.Z. , Schluter, J. , and Foster, K.R. (2015) The ecology of the microbiome: networks, competition, and stability. Science 350: 663–666. 26542567 10.1126/science.aad2602

[emi16031-bib-0013] de Vries, F.T. , Griffiths, R.I. , Bailey, M. , Craig, H. , Girlanda, M. , Gweon, H.S. , *et al*. (2018) Soil bacterial networks are less stable under drought than fungal networks. Nat Commun 9: 1–2. 30072764 10.1038/s41467-018-05516-7 PMC6072794

[emi16031-bib-0014] de Vries, F.T. , Griffiths, R.I. , Knight, C.G. , Nicolitch, O. , and Williams, A. (2020) Harnessing rhizosphere microbiomes for drought‐resilient crop production. Science 368: 270–274. 32299947 10.1126/science.aaz5192

[emi16031-bib-0015] Delgado‐Baquerizo, M. , Reich, P.B. , Trivedi, C. , Eldridge, D.J. , Abades, S. , Alfaro, F.D. , *et al*. (2020) Multiple elements of soil biodiversity drive ecosystem functions across biomes. Nat Ecol Evol 4: 210–220. 32015427 10.1038/s41559-019-1084-y

[emi16031-bib-0016] Durán, P. , Thiergart, T. , Garrido‐Oter, R. , Agler, M. , Kemen, E. , Schulze‐Lefert, P. , and Hacquard, S. (2018) Microbial interkingdom interactions in roots promote Arabidopsis survival. Cell 175: 973–983. 30388454 10.1016/j.cell.2018.10.020 PMC6218654

[emi16031-bib-0017] Edgar, R.C. (2010) Search and clustering orders of magnitude faster than BLAST. Bioinformatics hernan 26: 2460–2461. 10.1093/bioinformatics/btq461 20709691

[emi16031-bib-0018] Edgar, R.C. (2013) UPARSE: highly accurate OTU sequences from microbial amplicon reads. Nat Met 10: 996–998. 10.1038/nmeth.2604 23955772

[emi16031-bib-0019] Edgar, R.C. (2016) UNOISE2: improved error‐correction for Illumina 16S and ITS amplicon sequencing. BioRxiv 1: 081257.

[emi16031-bib-0020] Fierer, N. (2017) Embracing the unknown: disentangling the complexities of the soil microbiome. Nat Rev Microbiol 15: 579–590. 28824177 10.1038/nrmicro.2017.87

[emi16031-bib-0021] Fiore‐Donno, A.M. , Richter‐Heitmann, T. , Degrune, F. , Dumack, K. , Regan, K.M. , Marhan, S. , *et al*. (2019) Functional traits and spatio‐temporal structure of a major group of soil protists (Rhizaria: Cercozoa) in a temperate grassland. Front Microbiol 10: 1332. 31244819 10.3389/fmicb.2019.01332 PMC6579879

[emi16031-bib-0022] Fitzpatrick, C.R. , Copeland, J. , Wang, P.W. , Guttman, D.S. , Kotanen, P.M. , and Johnson, M.T. (2018) Assembly and ecological function of the root microbiome across angiosperm plant species. Proc Natl Acad Sci USA 115: E1157–E1165. 29358405 10.1073/pnas.1717617115 PMC5819437

[emi16031-bib-0023] Friedman, J. , and Alm, E.J. (2012) Inferring correlation networks from genomic survey data. PLoS Comput Biol 8: e1002687. 23028285 10.1371/journal.pcbi.1002687 PMC3447976

[emi16031-bib-0024] Fuchslueger, L. , Bahn, M. , Hasibeder, R. , Kienzl, S. , Fritz, K. , Schmitt, M. , *et al*. (2016) Drought history affects grassland plant and microbial carbon turnover during and after a subsequent drought event. J Ecol 104: 1453–1465. 27609992 10.1111/1365-2745.12593 PMC4996329

[emi16031-bib-0025] Gao, Z. , Karlsson, I. , Geisen, S. , Kowalchuk, G. , and Jousset, A. (2019) Protists: puppet masters of the rhizosphere microbiome. Trends Plant Sci 24: 165–176. 30446306 10.1016/j.tplants.2018.10.011

[emi16031-bib-0026] Geisen, S. , Mitchell, E.A. , Adl, S. , Bonkowski, M. , Dunthorn, M. , Ekelund, F. , *et al*. (2018) Soil protists: a fertile frontier in soil biology research. FEMS Microbiol Rev 42: 293–323. 29447350 10.1093/femsre/fuy006

[emi16031-bib-0027] Guillou, L. , Bachar, D. , Audic, S. , Bass, D. , Berney, C. , Bittner, L. , *et al*. (2012) The Protist ribosomal reference database (PR2): a catalog of unicellular eukaryote small sub‐unit rRNA sequences with curated taxonomy. Nucl Acids Res 41: D597–D604. 23193267 10.1093/nar/gks1160 PMC3531120

[emi16031-bib-0028] Guo, S. , Xiong, W. , Hang, X. , Gao, Z. , Jiao, Z. , Liu, H. , *et al*. (2021) Protists as main indicators and determinants of plant performance. Microbiome 9: 1. 33743825 10.1186/s40168-021-01025-w PMC7981826

[emi16031-bib-0029] Hamonts, K. , Trivedi, P. , Garg, A. , Janitz, C. , Grinyer, J. , Holford, P. , *et al*. (2018) Field study reveals core plant microbiota and relative importance of their drivers. Environ Microbiol 20: 124–140. 29266641 10.1111/1462-2920.14031

[emi16031-bib-0030] Harder, C.B. , Rønn, R. , Brejnrod, A. , Bass, D. , Al‐Soud, W.A. , and Ekelund, F. (2016) Local diversity of heathland Cercozoa explored by in‐depth sequencing. ISME J 10: 2488–2497. 26953604 10.1038/ismej.2016.31 PMC5030685

[emi16031-bib-0129] Hernandez, D.J., David, A.S., Menges, E.S., Searcy, C.A., and Afkhami, M.E. (2021). Environmental stress destabilizes microbial networks. *ISME J* **15**: 1722‐1734. 10.1038/s41396-020-00882-x PMC8163744 33452480

[emi16031-bib-0032] Leff, J.W. (2017) mctoolsr: Microbial Community Data Analysis Tools. R package version 0.1.1.2.

[emi16031-bib-0033] Martin, M. (2011) Cutadapt removes adapter sequences from high‐throughput sequencing reads. EMBnet J 17: 10–12.

[emi16031-bib-0034] Naylor, D. , and Coleman‐Derr, D. (2018) Drought stress and root‐associated bacterial communities. Front Plant Sci 9: 2223. 10.3389/fpls.2017.02223 PMC5767233 29375600

[emi16031-bib-0035] Naylor, D. , DeGraaf, S. , Purdom, E. , and Coleman‐Derr, D. (2017) Drought and host selection influence bacterial community dynamics in the grass root microbiome. ISME J 11: 2691–2704. 28753209 10.1038/ismej.2017.118 PMC5702725

[emi16031-bib-0036] Nilsson, R.H. , Larsson, K.H. , Taylor, A.F. , Bengtsson‐Palme, J. , Jeppesen, T.S. , Schigel, D. , *et al*. (2019) The UNITE database for molecular identification of fungi: handling dark taxa and parallel taxonomic classifications. Nucl Acids Res 47: D259–D264. 30371820 10.1093/nar/gky1022 PMC6324048

[emi16031-bib-0037] Ochoa‐Hueso, R. , Collins, S.L. , Delgado‐Baquerizo, M. , Hamonts, K. , Pockman, W.T. , Sinsabaugh, R.L. , *et al*. (2018) Drought consistently alters the composition of soil fungal and bacterial communities in grasslands from two continents. Glob Change Biol 24: 2818–2827. 10.1111/gcb.14113 29505170

[emi16031-bib-0038] Oliverio, A.M. , Geisen, S. , Delgado‐Baquerizo, M. , Maestre, F.T. , Turner, B.L. , and Fierer, N. (2020) The global‐scale distributions of soil protists and their contributions to belowground systems. Sci Adv 6: eaax8787. 32042898 10.1126/sciadv.aax8787 PMC6981079

[emi16031-bib-0039] Pruesse, E. , Quast, C. , Knittel, K. , Fuchs, B.M. , Ludwig, W. , Peplies, J. , and Glöckner, F.O. (2007) SILVA: a comprehensive online resource for quality checked and aligned ribosomal RNA sequence data compatible with ARB. Nucl Acids Res 35: 7188–7196. 17947321 10.1093/nar/gkm864 PMC2175337

[emi16031-bib-0040] Puértolas, J. , Larsen, E.K. , Davies, W.J. , and Dodd, I.C. (2017) Applying ‘drought’ to potted plants by maintaining suboptimal soil moisture improves plant water relations. J Exp Botany 68: 2413–2424. 28419363 10.1093/jxb/erx116 PMC5447888

[emi16031-bib-0041] R Core Team . (2020) R: A Language and Environment for Statistical Computing. Vienna, Austria: R Foundation for Statistical Computing.

[emi16031-bib-0042] Rizaludin, M.S. , Stopnisek, N. , Raaijmakers, J.M. , and Garbeva, P. (2021) The chemistry of stress: understanding the ‘cry for help’ of plant roots. Metabolites 11: 357. 34199628 10.3390/metabo11060357 PMC8228326

[emi16031-bib-0043] Rodriguez, R. , and Durán, P. (2020) Natural holobiome engineering by using native extreme microbiome to counteract the climate change effects. Front Bioeng Biotech 8: 568. 10.3389/fbioe.2020.00568 PMC7287022 32582678

[emi16031-bib-0044] Rolfe, S.A. , Griffiths, J. , and Ton, J. (2019) Crying out for help with root exudates: adaptive mechanisms by which stressed plants assemble health‐promoting soil microbiomes. Curr Opin Microbiol 49: 73–82. 31731229 10.1016/j.mib.2019.10.003

[emi16031-bib-0045] Santos‐Medellín, C. , Edwards, J. , Liechty, Z. , Nguyen, B. , and Sundaresan, V. (2017) Drought stress results in a compartment‐specific restructuring of the rice root‐associated microbiomes. MBio 8: e00764–e00717. 28720730 10.1128/mBio.00764-17 PMC5516253

[emi16031-bib-0046] Santos‐Medellín, C. , Liechty, Z. , Edwards, J. , Nguyen, B. , Huang, B. , Weimer, B.C. , and Sundaresan, V. (2021) Prolonged drought imparts lasting compositional changes to the rice root microbiome. Nat Plants 7: 1065–1077. 34294907 10.1038/s41477-021-00967-1

[emi16031-bib-0047] Schmitt, A. , and Glaser, B. (2011) Organic matter dynamics in a temperate forest soil following enhanced drying. Soil Biol Biochem 43: 478–489.

[emi16031-bib-0048] Segata, N. , Izard, J. , Waldron, L. , Gevers, D. , Miropolsky, L. , Garrett, W.S. , and Huttenhower, C. (2011) Metagenomic biomarker discovery and explanation. Genome Biol 12: 1–8. 10.1186/gb-2011-12-6-r60 PMC3218848 21702898

[emi16031-bib-0049] Simmons, T. , Caddell, D.F. , Deng, S. , and Coleman‐Derr, D. (2018) Exploring the root microbiome: extracting bacterial community data from the soil, rhizosphere, and root endosphere. J Vis Exp 135: 57561. 10.3791/57561 PMC6101100 29782021

[emi16031-bib-0050] Singh, S. , Mayes, M.A. , Shekoofa, A. , Kivlin, S.N. , Bansal, S. , and Jagadamma, S. (2021) Soil organic carbon cycling in response to simulated soil moisture variation under field conditions. Sci Rep 11: 1–3. 34035390 10.1038/s41598-021-90359-4 PMC8149407

[emi16031-bib-0051] Stefan, G. , Cornelia, B. , Jörg, R. , and Michael, B. (2014) Soil water availability strongly alters the community composition of soil protists. Pedobiologia 57: 205–213.

[emi16031-bib-0052] Stoeck, T. , Bass, D. , Nebel, M. , Christen, R. , Jones, M.D. , Breiner, H.W. , and Richards, T.A. (2010) Multiple marker parallel tag environmental DNA sequencing reveals a highly complex eukaryotic community in marine anoxic water. Mol Ecol 19: 21–31. 20331767 10.1111/j.1365-294X.2009.04480.x

[emi16031-bib-0053] Sun, A. , Jiao, X.Y. , Chen, Q. , Trivedi, P. , Li, Z. , Li, F. , *et al*. (2021) Fertilization alters protistan consumers and parasites in crop‐associated microbiomes. Environ Microbiol 23: 2169–2183. 33400366 10.1111/1462-2920.15385

[emi16031-bib-0054] Trivedi, P. , Batista, B.D. , Bazany, K.E. , and Singh, B.K. (2022) Plant–microbiome interactions under a changing world: responses, consequences and perspectives. New Phytol. doi:10.1111/nph.18016 35118660

[emi16031-bib-0055] Trivedi, P. , Delgado‐Baquerizo, M. , Trivedi, C. , Hamonts, K. , Anderson, I.C. , and Singh, B.K. (2017) Keystone microbial taxa regulate the invasion of a fungal pathogen in agro‐ecosystems. Soil Biol Biochem 111: 10–14.

[emi16031-bib-0056] Trivedi, P. , Delgado‐Baquerizo, M. , Trivedi, C. , Hu, H. , Anderson, I.C. , Jeffries, T.C. , *et al*. (2016) Microbial regulation of the soil carbon cycle: evidence from gene–enzyme relationships. ISME J 10: 2593–2604. 27168143 10.1038/ismej.2016.65 PMC5113853

[emi16031-bib-0057] Trivedi, P. , Leach, J.E. , Tringe, S.G. , Sa, T. , and Singh, B.K. (2020) Plant–microbiome interactions: from community assembly to plant health. Nat Rev Microbiol 18: 607–621. 32788714 10.1038/s41579-020-0412-1

[emi16031-bib-0058] Trivedi, P. , Mattupalli, C. , Eversole, K. , and Leach, J.E. (2021) Enabling sustainable agriculture through understanding and enhancement of microbiomes. New Phytol 230: 2129–2147. 33657660 10.1111/nph.17319

[emi16031-bib-0059] Veach, A.M. , Chen, H. , Yang, Z.K. , Labbe, A.D. , Engle, N.L. , Tschaplinski, T.J. , *et al*. (2020) Plant hosts modify belowground microbial community response to extreme drought. Msystems 5: e00092–e00020. 32606021 10.1128/mSystems.00092-20 PMC7329318

[emi16031-bib-0060] Watts, S.C. , Ritchie, S.C. , Inouye, M. , and Holt, K.E. (2019) FastSpar: rapid and scalable correlation estimation for compositional data. Bioinformatics 35: 1064–1066. 30169561 10.1093/bioinformatics/bty734 PMC6419895

[emi16031-bib-0061] Xie, J. , Wang, X. , Xu, J. , Xie, H. , Cai, Y. , Liu, Y. , and Ding, X. (2021) Strategies and structure feature of the aboveground and belowground microbial community respond to drought in wild rice (*Oryza longistaminata*). Rice 14: 1–7. 34495440 10.1186/s12284-021-00522-8 PMC8426455

[emi16031-bib-0062] Xiong, C. , He, J.Z. , Singh, B.K. , Zhu, Y.G. , Wang, J.T. , Li, P.P. , *et al*. (2021a) Rare taxa maintain the stability of crop mycobiomes and ecosystem functions. Environ Microbiol 23: 1907–1924. 32996254 10.1111/1462-2920.15262

[emi16031-bib-0063] Xiong, C. , Zhu, Y.G. , Wang, J.T. , Singh, B.K. , Han, L.L. , Shen, J.P. , *et al*. (2021b) Host selection shapes crop microbiome assembly and network complexity. New Phytol 229: 1091–1104. 32852792 10.1111/nph.16890

[emi16031-bib-0064] Xiong, W. , Jousset, A. , Guo, S. , Karlsson, I. , Zhao, Q. , Wu, H. , *et al*. (2018) Soil protist communities form a dynamic hub in the soil microbiome. ISME J 12: 634–638. 29028001 10.1038/ismej.2017.171 PMC5776453

[emi16031-bib-0065] Xu, L. , and Coleman‐Derr, D. (2019) Causes and consequences of a conserved bacterial root microbiome response to drought stress. Curr Opin Microbiol 49: 1–6. 31454709 10.1016/j.mib.2019.07.003

[emi16031-bib-0066] Xu, L. , Naylor, D. , Dong, Z. , Simmons, T. , Pierroz, G. , Hixson, K.K. , *et al*. (2018) Drought delays development of the sorghum root microbiome and enriches for monoderm bacteria. Proc Natl Acad Sci U S A 115: E4284–E4293. 29666229 10.1073/pnas.1717308115 PMC5939072

[emi16031-bib-0067] Yuan, M.M. , Guo, X. , Wu, L. , Zhang, Y. , Xiao, N. , Ning, D. , *et al*. (2021) Climate warming enhances microbial network complexity and stability. Nat Clim Change 11: 343–348.

[emi16031-bib-0068] Yuste, J.C. , Penuelas, J. , Estiarte, M. , Garcia‐Mas, J. , Mattana, S. , Ogaya, R. , *et al*. (2011) Drought‐resistant fungi control soil organic matter decomposition and its response to temperature. Glob Change Biol 17: 1475–1486.

[emi16031-bib-0069] Zhang, J. , Liu, S. , Liu, C. , Wang, H. , Luan, J. , Liu, X. , *et al*. (2021) Soil bacterial and fungal richness and network exhibit different responses to long‐term throughfall reduction in a warm‐temperate oak forest. Forests 12: 165.

[emi16031-bib-0070] Zhou, J. , Deng, Y. , Luo, F. , He, Z. , and Yang, Y. (2011) Phylogenetic molecular ecological network of soil microbial communities in response to elevated CO_2_ . MBio 2: e00122–e00111. 21791581 10.1128/mBio.00122-11 PMC3143843

