# Supplementary material for: Water deficit affects inter‐kingdom microbial connections in plant rhizosphere
Source: Environ Microbiol. 2022 May 17;24(8):3722–34. doi: 10.1111/1462-2920.16031 (PMC9545320; doi:10.1111/1462-2920.16031)
Supplement: Supplementary file 1 — Appendix S1: Supporting Information [file EMI-24-3722-s001.docx]

**Supplementary Information:**

**
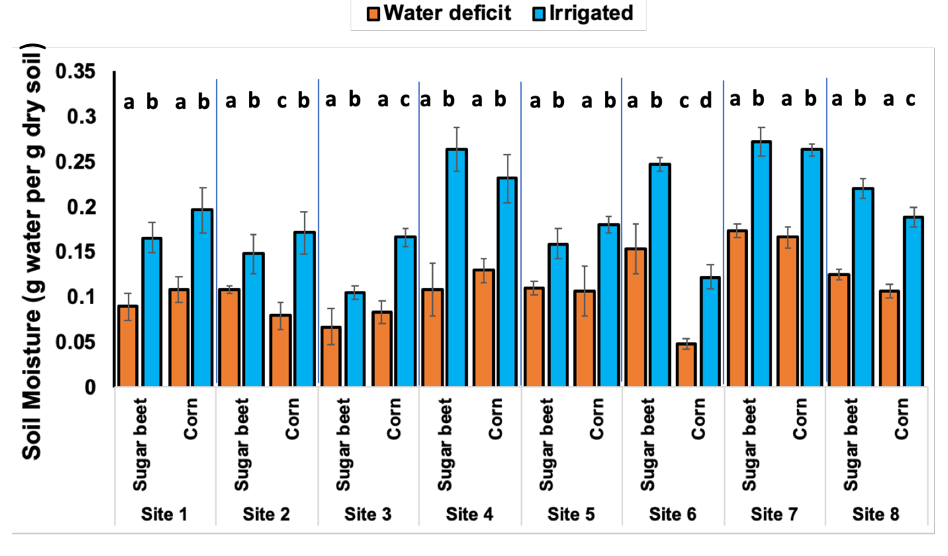
**

**Supplementary Fig. 1:** Differences in soil volumetric water content (VWC, %) between irrigated (blue) and water deficit (brown) treatments for sugar beet and corn at different sites. Different letters mean statistically significant differences at *p* < 0.05 within each site. Error bars indicate Standard error.


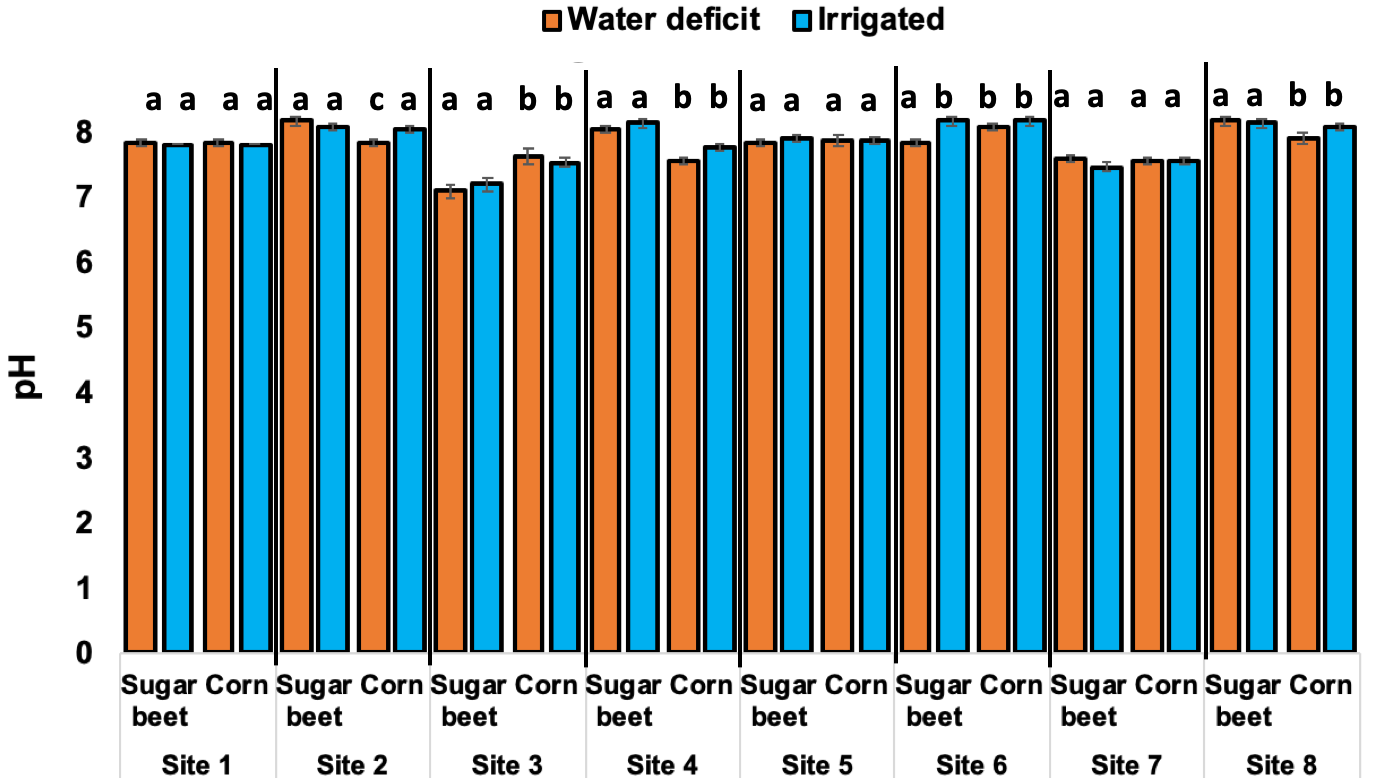


**Supplementary Fig. 2:** Differences in soil pH between irrigated (blue) and water deficit (brown) treatments for sugar beet and corn at different sites. Different letters mean statistically significant differences at p < 0.05 within each site. Error bars indicate Standard error.

**
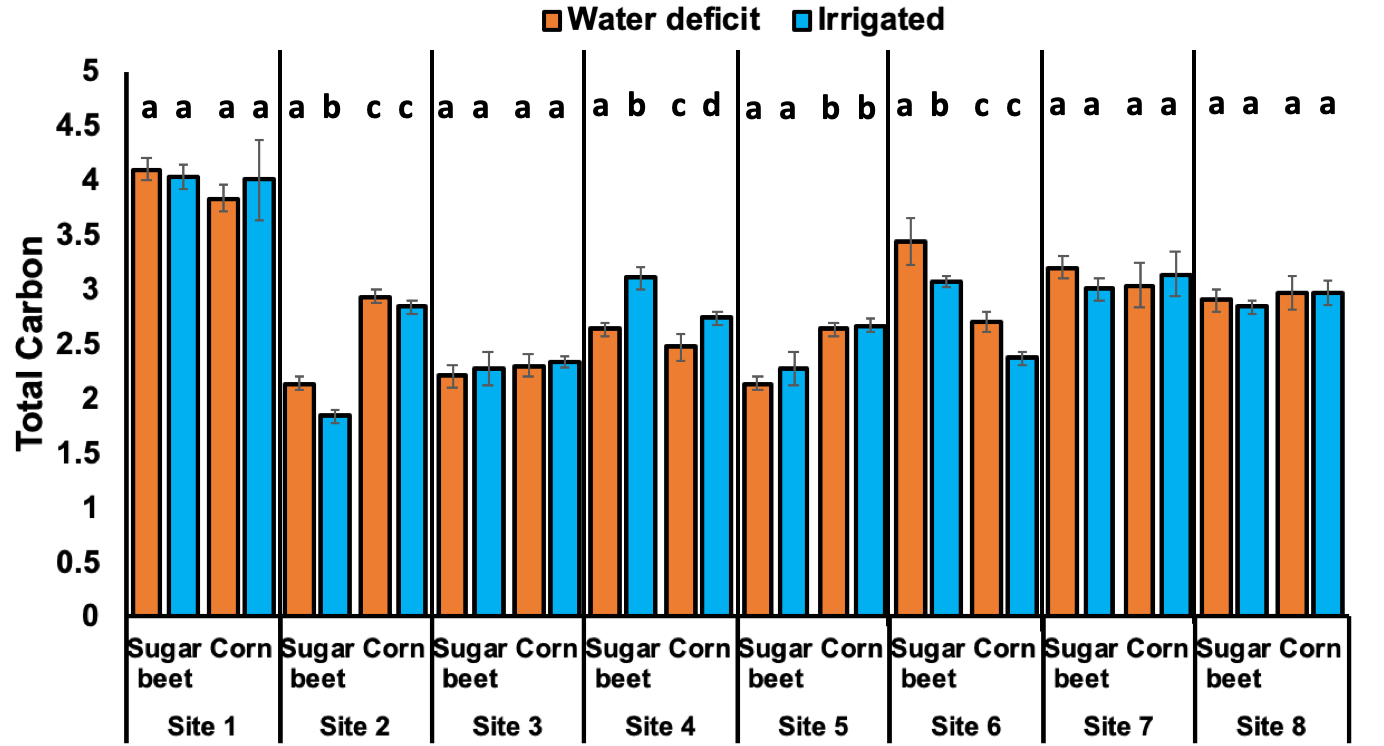
**

**Supplementary Fig. 3:** Differences in total carbon between irrigated (blue) and water deficit (brown) treatments for sugar beet and corn at different sites. Different letters mean statistically significant differences at *p* < 0.05 within each site. Error bars indicate Standard error.


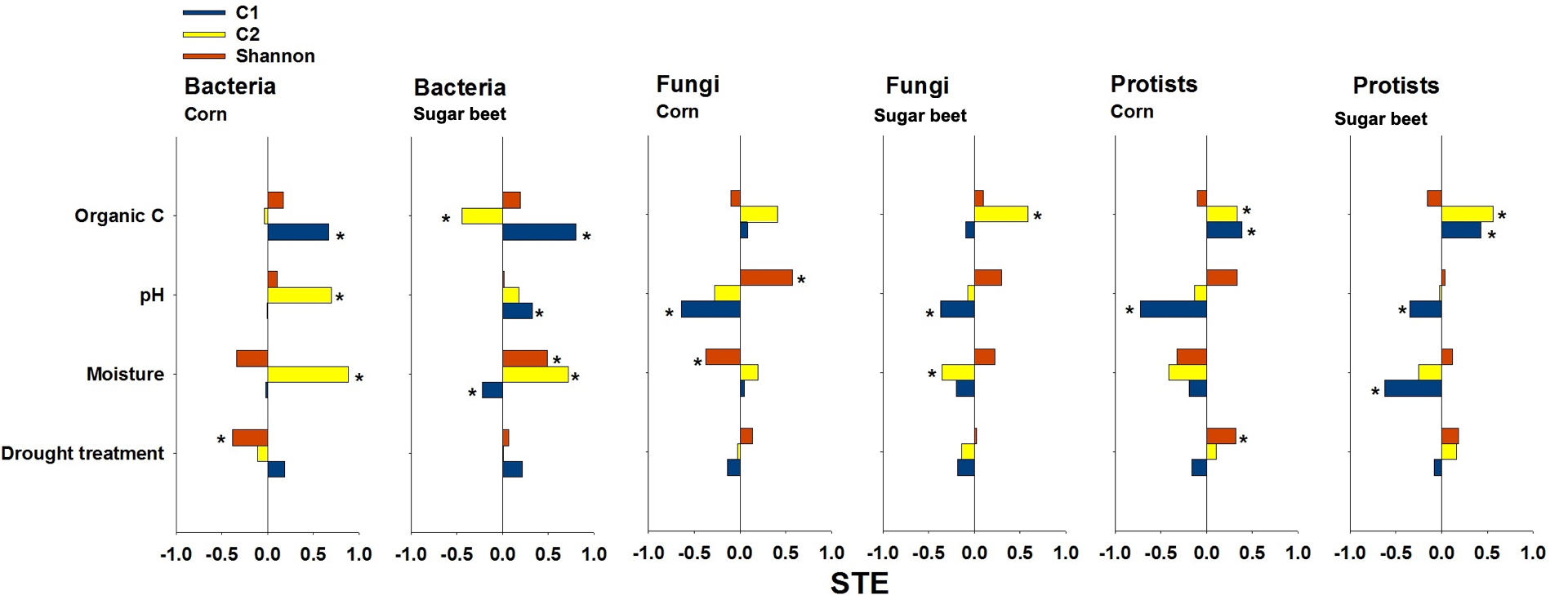


**Supplementary Fig. 4:** Standardized total effects (STE) showing direct plus indirect effects of organic C, pH, soil moisture and treatment on the community composition (C1 and C2) and Shannon diversity of bacteria, fungi, and protist in the rhizosphere of corn and sugar beet. STEs are derived from the structural equation model presented in Fig. 2.


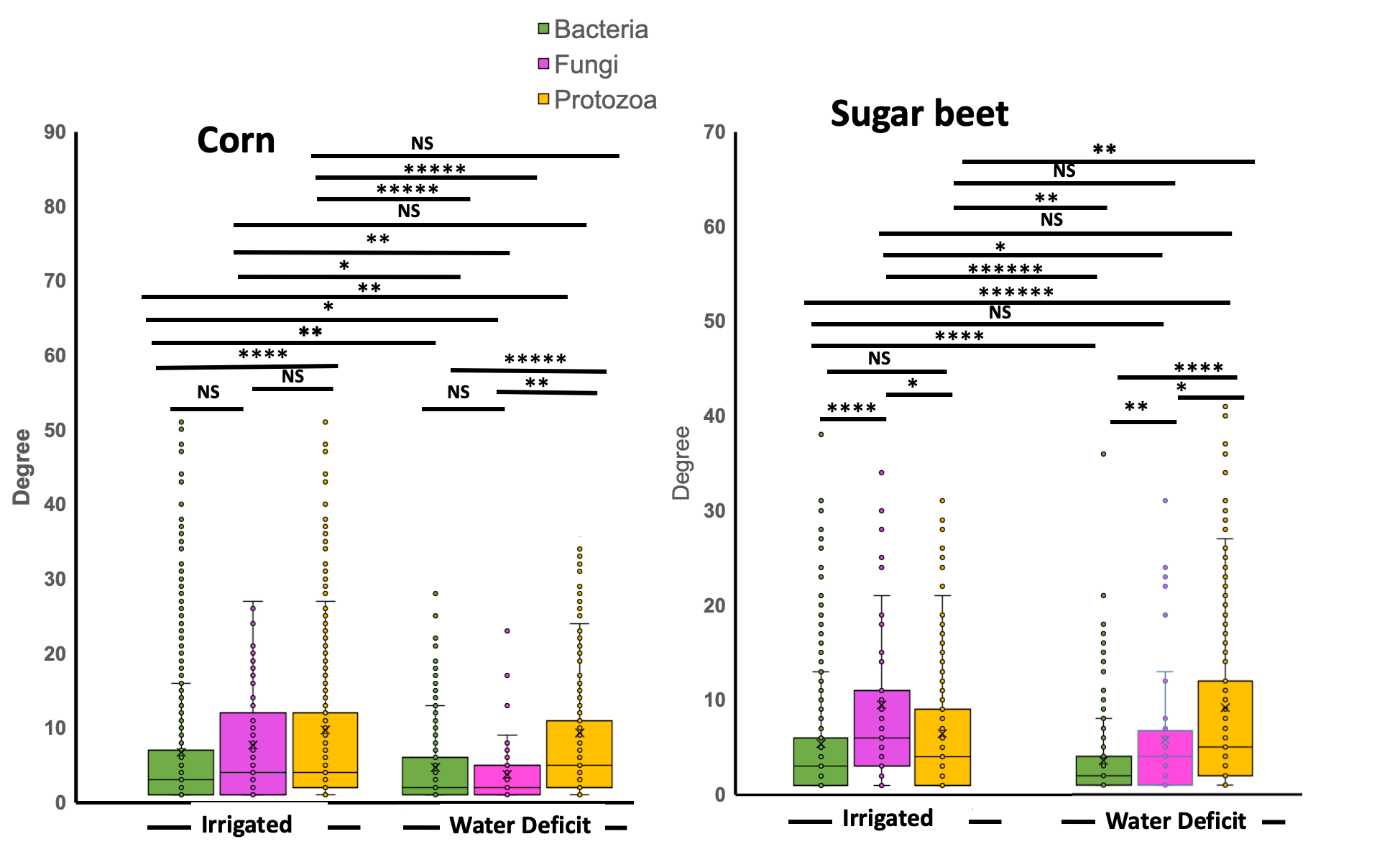


**Supplementary Fig. 5:** Degree values of bacteria, fungi, and protist taxa in irrigated and water deficit networks for corn and sugar beet. The significance of difference was determined by nonparametric Kruskal–Wallis test. *, **, ***, ****, ***** represent significance at *p* values of < 0.05, 0.005, 0.0005, 0.00005, and 0.000005, respectively. NS = non-significant.


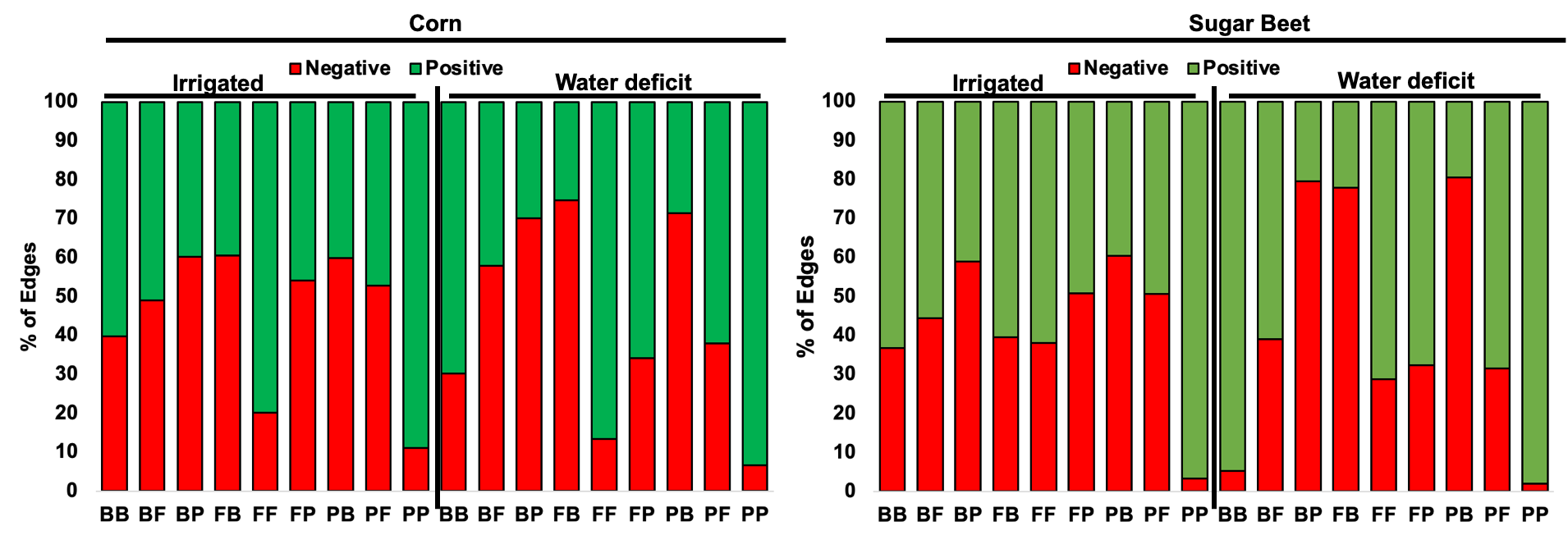


**Supplementary Fig. 6:** Percentage of edges showing positive (green) or negative (red) correlations in the irrigated and water deficit microbial rhizosphere network of corn and sugar beet. B, bacteria; F, fungi; P, protists.

Supplementary Table 1. Site information.

| **Site ID** | **State** | **Latitude** | **Longitude** |
| --- | --- | --- | --- |
| NE 4 | Nebraska | 42.278668 | -102.911604 |
| NE 7 | Nebraska | 41.843778 | -103.595356 |
| CO 1 | Colorado | 40.638932 | -105.015802 |
| CO 3 | Colorado | 40.30281 | -104.925196 |
| CO 8 | Colorado | 40.24657 | -104.099522 |
| CO 10 | Colorado | 40.287572 | -103.641678 |
| MT 1 | Montana | 45.43407 | -108.88046 |
| MT 2 | Montana | 45.72424 | -108.63317 |
